# Curcumin-Rich Curry Consumption and Neurocognitive Function from 4.5-Year Follow-Up of Community-Dwelling Older Adults (Singapore Longitudinal Ageing Study)

**DOI:** 10.3390/nu14061189

**Published:** 2022-03-11

**Authors:** Tze Pin Ng, Ma Shwe Zin Nyunt, Qi Gao, Xinyi Gwee, Denise Qian Ling Chua, Keng Bee Yap

**Affiliations:** 1 Department of Psychological Medicine, Yong Loo Lin School of Medicine, National University of Singapore, Singapore 119228, Singapore; pcmgxy@nus.edu.sg (X.G.); pcmdcql@nus.edu.sg (D.Q.L.C.); 2 Geriatric Education and Research Institute, Singapore 768024, Singapore; 3 Office of the Senior Deputy President and Provost, National University of Singapore, Singapore 119077, Singapore; szn@nus.edu.sg; 4 National Public Health and Epidemiology Unit, National Centre for Infectious Diseases, Singapore 308442, Singapore; qi_gao@ncid.sg; 5 Department of Medicine, Ng Teng Fong General Hospital, Singapore 609606, Singapore; keng_bee_yap@nuhs.edu.sg

**Keywords:** turmeric, cognition, longitudinal

## Abstract

The potential neurocognition protective effects of dietary curcumin in curry consumed with food was investigated in this study of 2734 community-dwelling adults (aged ≥ 55, mean ± SD: 65.9 ± 7.4). We analyzed longitudinal data of baseline curry consumption (“never or rarely”, “occasionally”: <once a month, “often”: >once a month and <once a week, “very often”: >once a week or daily) and baseline and 4.5-year follow-up cognitive function in mixed model analyses controlling for confounding risk factors. Significant between-exposure differences were found for Digit Span-Backward (DS-B), Verbal Fluency-Animals (VF-A) and Block Design (BD). Compared to “never or rarely” consumption, “very often” and “often” consumptions were associated with higher DS-B performance; “very often”—with higher VF-A, and “occasional”, “often” and “very often” consumptions—with higher BD: Cohen’s d: from 0.130 to 0.186. Among participants with cardiometabolic and cardiac diseases (CMVD), curry consumption was associated with significantly higher DS-B and VF-A. Among CMVD-free participants, curry consumption was associated with significantly higher DS-B, VF-A and BD: Cohen’s d: from 0.098 to 0.305. The consumption of dietary curcumin was associated with the maintenance over time of higher functioning on attention, short-term working memory, visual spatial constructional ability, language and executive function among community-dwelling older Asian adults.

## 1. Introduction

Curcumin is a polyphenolic compound isolated from the roots of *Curcuma longa*, from which turmeric is prepared and used widely as a coloring agent, food additive and traditional Indian and Chinese medicine. For decades, curcumin and related bioactive curcuminoids (demethoxycurcumin and bisdemethoxycurcumin) have been the center of interest of scientific studies worldwide into their therapeutic potentials. Numerous laboratory studies have documented its antioxidant, anti-inflammatory, antimicrobial, antiviral, antineoplastic and antiaging activities [1,2,3]. Curcumin has low bioavailability due to its poor aqueous solubility, low intestinal absorption, low tissue distribution, rapid metabolism and systemic clearance [4]. Hence, various formulations of curcumin, including nanoparticles and liposomal encapsulation, are used in over 100 completed and ongoing clinical trials to investigate its therapeutic potential for chronic diseases, including cancers, diabetes, obesity, cardiovascular, pulmonary, neurological and autoimmune diseases [1].

Curcumin is actively being investigated for its potential neuroprotective benefits. Accelerated cognitive decline and dementia are principally the result of brain neurodegenerative disease due to underlying β amyloid (Aβ) and tau pathology, cerebral microvascular disease and neuroinflammation. In vitro and in vivo studies show that curcumin crosses the blood–brain barrier and can bind to β amyloid (Aβ) and tau-inhibiting Aβ aggregation and modulate tau processing [5,6,7,8,9]. Animal studies show that curcumin decreases Aβ deposition and plaque load in the brain of transgenic mouse models of AD [10,11]. Experimental studies have also demonstrated that curcumin possesses beneficial antiplatelet, antidiabetic and cardioprotective activities ameliorating cardiac hypertrophy and chronic heart failure [12,13,14,15,16,17,18,19]. On top of the known anti-inflammatory activities of curcumin attenuating neuroinflammation, this experimental evidence strongly suggests that curcumin has the potential to prevent accelerated cognitive decline in humans. Several animal studies have shown that curcumin improves cognitive function [20,21]. Randomized controlled trials in humans are limited but suggest that curcumin could be safely and effectively used to slow cognitive decline [22,23,24,25,26,27].

We have previously reported preliminary findings from an observational cross-sectional study that older individuals who consumed greater amounts of curry in their meals showed a higher global cognitive performance score on the Mini-Mental State Examination (MMSE) compared to their counterparts who never or rarely consumed curry [28]. To our knowledge, there has not been any other naturalistic study that has investigated the potential cognitive benefits of dietary food source(s) of curcumin in humans. The main source of curcumin in the diet is almost exclusively from turmeric, the Indian curry spice, which is most commonly used in curry meals consumed by Asians in subcontinental India, Southeast and other parts of Asia. It is therefore pertinent to investigate whether the consumption of curcumin naturally in the diet is associated with enhanced cognitive functioning in older people who are vulnerable to the risk of developing dementia.

In this report, we describe the results of a larger prospective study in a separate cohort of community-dwelling middle-aged and older adults in the Singapore Longitudinal Ageing Study (SLAS) with longitudinal analysis of cognitive function assessed at baseline and 4.5-year follow-up.

## 2. Materials and Methods

*Study design and participants.* We analyzed data in two combined population cohorts in the Singapore Longitudinal Ageing Study (SLAS-1 and SLAS-2). SLAS-1 recruited 2804 participants in Southeast Singapore from Sep 2003 to Dec 2004, and SLAS-2 recruited 3270 participants in Southwest Singapore from Mar 2009 to Jun 2013. First wave follow-up visits and assessments were conducted at approximately 3–5 years after the baseline assessment, from Mar 2005 to Sep 2007 for SLAS-1, and Jan 2013–Aug 2018 for SLAS-2. Details of methodology of SLAS-1 and SLAS-2 cohorts have been described in earlier reports [29,30].

*Inclusion and exclusion criteria:* Singapore citizens or permanent residents in study locations who were aged ≥55 years at recruitment. We excluded individuals who were unable to participate due to severe physical or mental disability, those with stroke, traumatic brain injury and Parkinson’s disease or other brain neurodegenerative disorders.

*Ethics approval.* This study was approved by the National University of Singapore Institutional Review Board (Protocol Code: 04–140), in accordance with the relevant guidelines and regulations by the Declaration of Helsinki and the ethical principles in the Belmont Report. All participants gave written informed consent to participate in the study.

*Measurements.* Trained nurses visited participants’ homes to perform face-to-face questionnaire interviews, and clinical measurements were performed in a local study site center to collect an extensive range of sociodemographic, lifestyle, behavior, psychological, neurobehavioral, medical and other data at baseline. Among 6074 participants in the combined cohort, curry consumption data was available for 5226 participants. Neurocognitive performance tests were performed on a random 1-in-3 sample of SLAS participants, and 1-in-2 sample of SLAS2 participants. This resulted in 2846 participants with complete curry consumption data and data for at least one neurocognitive test. Among them, 95 participants with stroke and Parkinson’s disease were excluded from analysis in this study. The final analytical sample was *N* = 2751, including participants with baseline neurocognitive data varying in numbers between *N* = 1674 and *N* = 2734 for different tests. Follow-up neurocognitive test data were variously available for between 1202 and 1747 participants.

*Curry consumption.* Participants were asked at baseline and follow-up interviews how frequently they usually consumed curry in their meals: Never or rarely (less than once a year), occasionally (at least once/year to less than once/month), often (at least once/month to less than once/week, very often (at least once a week) or daily. Because of the small number of participants who reported daily consumption in this study, they were included in a combined category of “very often or daily”. Participants were also asked whether their curry consumption had remained mostly unchanged or have changed. Only strictly consistent responses at baseline and follow-up interviews were used to assign them unequivocally to the highest and the lowest consumption category. Participants who consistently reported “occasional” or “often” consumption frequencies at baseline and follow-up were also assigned as such to the appropriate category. For modestly inconsistent responses (differing by one response category at baseline and follow-up), the higher consumption response was used to assign to the nearest “occasional” or “often” categories. For more widely inconsistent responses, we used the average response score to assign them to the nearest response category, either “occasional” or “often”.

*Neurocognitive assessments.* Global cognition was assessed using a locally translated version of the Mini Mental State Examination (MMSE) which was previously validated for use among Chinese and non-Chinese (Malay, Indian and others) Singaporean older adults [31]. A comprehensive battery of neurocognitive tests was used for assessment of cognitive domains, including verbal learning and memory: Rey Auditory Verbal Learning Test-Delayed Recall (RAVLT-DR), visual memory: Visual Reproduction-Delayed Recall (VR-DR), attention and working memory: Digit Span Forward (DS-F) and Backward (DS-B), language: Categorical Verbal Fluency-Animals (VF-A), executive function, visual, spatial and constructional ability: Block Design (BD), visual-motor tracking and mental flexibility: Trail Making Test A (TMT-A) and B (TMT-B). The assessment was administered by trained psychology research assistants in the participants’ habitual language: English, Mandarin, or dialects. The validation of the neuropsychological tests for use in Singaporean older adults and their normative values have been described previously [32]. The test performance results were standardized by transforming their raw scores into z-scores.

*Covariates.* Other measurements were made for potential confounding risk factors that included sex, age (single years), ethnicity (Chinese versus non-Chinese (Malay, Indian Other)), education (none, 1–6 years, >6 years), smoking (never, past smoker, current smoker), alcohol (≥once/week) and body mass index (BMI, kg/m^2^). Physical activity score, social activity score and productive activity score were calculated from the number and frequencies of usual participation in 18 different categories of physical, social and productive activities [29]. Central obesity was defined as waist circumference ≥80 cm in women and ≥90 cm in men [33]. Hypertension was assessed using the criteria of systolic blood pressure ≥140 mmHg and/or diastolic blood pressure ≥90 mmHg [34], or a self-report history of hypertension diagnosis and treatment, verified by recorded names of drugs shown on medication packages. Diabetes was assessed using fasting blood glucose concentrations of ≥5.6 mmol/L [35] or a self-report history of diabetes diagnosis and treatment. Dyslipidemia was defined as either triglyceride levels of ≥1.7 mmol/L or high-density lipoprotein cholesterol <1 mmol/L in men and <1.3 mmol/L in women [36]. The presence of cardiovascular disease was defined by a self-report history of stroke, myocardial infarct, or heart failure. Depressive symptoms were assessed by the locally translated and validated 15-item Geriatric Depression Scale (GDS) [37].

*Statistical analysis.* Categorical variables are presented as numbers (percentages), and continuous variables are presented as means (SD). Differences between curry exposure groups were examined using ANOVA for continuous variables, and chi-square test for categorical variables. Between-group differences over time for neurocognitive performances were analyzed using mixed-effect model with curry group (≥1 x/week vs. <1 x/week), time (baseline and follow-up) and their interactions as fixed effects and subject as random effect. Maximum likelihood estimation was used to analyze missing data for neurocognitive performance. To control for confounding by risk factors, the mixed models included as additional covariates of age, sex, ethnicity (Chinese versus non-Chinese), educational level, physical, social and productive activity scores, smoking (nonsmoker, ex-smoker, current smoker), alcohol (never, <once/week, ≥once/week), central obesity, hypertension, high fasting blood glucose or diabetes, high triglyceride, low HDL-cholesterol, cardiac diseases and GDS score. Effect size was estimated using Cohen’s d statistic. All statistical analyses were performed using IBM SPSS Statistics version 27, and a value of *p* < 0.05 was considered significant.

## 3. Results

The mean (SD) age of the study participants was 65.9 (7.4), and 63.6% were women. Participants who reported higher levels of curry consumption were more likely to be younger, men, of non-Chinese (Malay, Indian) ethnicity, better educated, had higher levels of productive (mentally stimulating) activity, more central obesity, and lower GDS depression scores (Table 1).

As shown in Supplementary Table S1, these known or potential risk factors were significantly associated with various neurocognitive performance. In particular, Chinese ethnicity was significantly associated with all neurocognitive test performance; age was significantly associated with MMSE, VR-DR, TMT-A, TMT-B and BD; education was significantly associated with MMSE, DS-F, DS-B, RAVLT-DR, VR-DR and VF-A; productive activity was associated with MMSE, RAVLT, VR-DR, TMT-A and BD; central obesity was associated with MMSE, VR-DR; and GDS with MMSE, RAVLT, VR-DR and BD.

**Figure 1:**
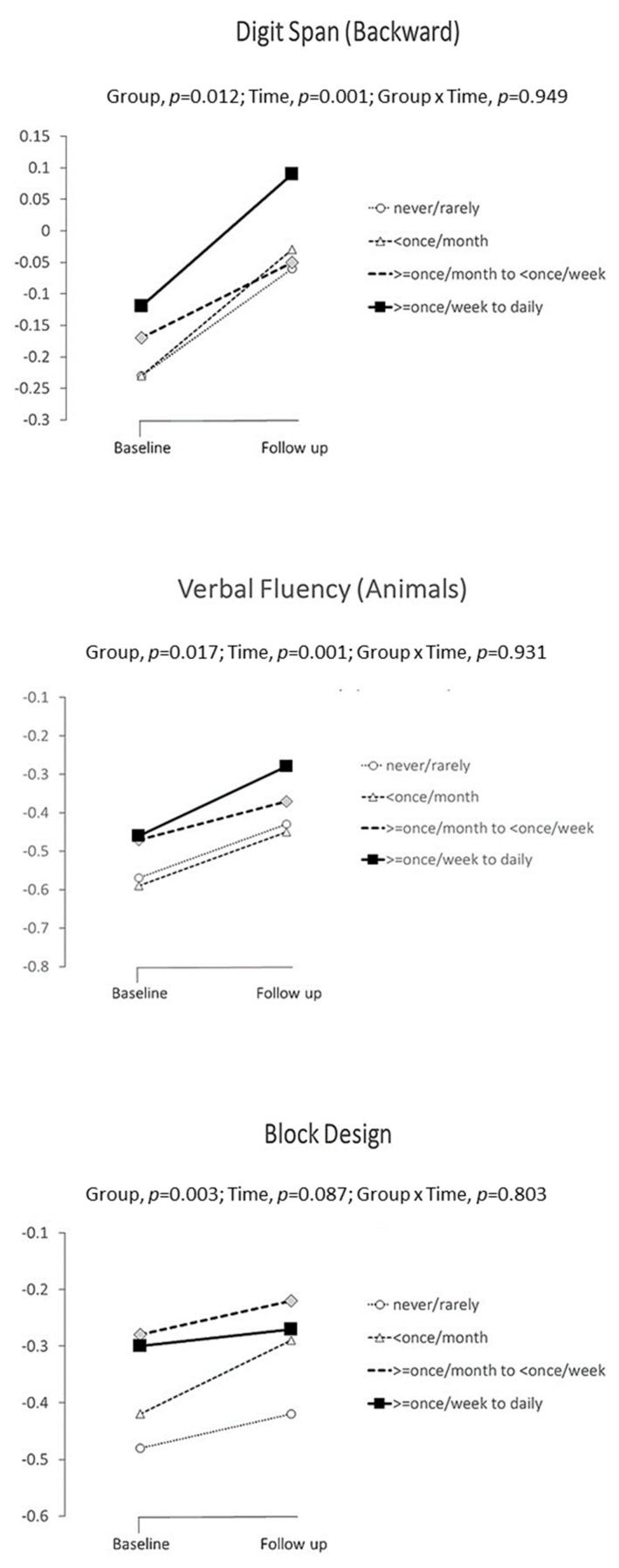
Significant associations between curry consumption and Digit Span (Backward), Verbal Fluency (Animals) and Block Design.

Increasing levels of curry consumption were significantly associated with better crude estimates of mean neurocognitive performance scores at both baseline and follow-up (Table 2) for MMSE, Digit Span-Backward, Verbal Fluency-Animals, Trail-Making Tests A and B and Block Design. In mixed model analyses, (Table 3 and Figure 1) controlling for known or potential confounding risk factors, significant between-group differences were shown for Digit Span (Backward), Verbal Fluency (Animals) and Block Design. Pairwise comparisons showed that compared to “never or rarely” consumption, “very often” and “often” consumptions were associated with significantly higher levels of Digit Span-Backward; “very often” was associated with significantly higher performance on Verbal Fluency-Animals, and “occasional”, “often” and “very often” consumptions were associated with significantly higher performance on Block Design. The estimated Cohen’s d for significantly enhanced neurocognitive functions ranged from 0.130 (Digit Span-Backward) to 0.186 (Block Design).

**Figure 2:**
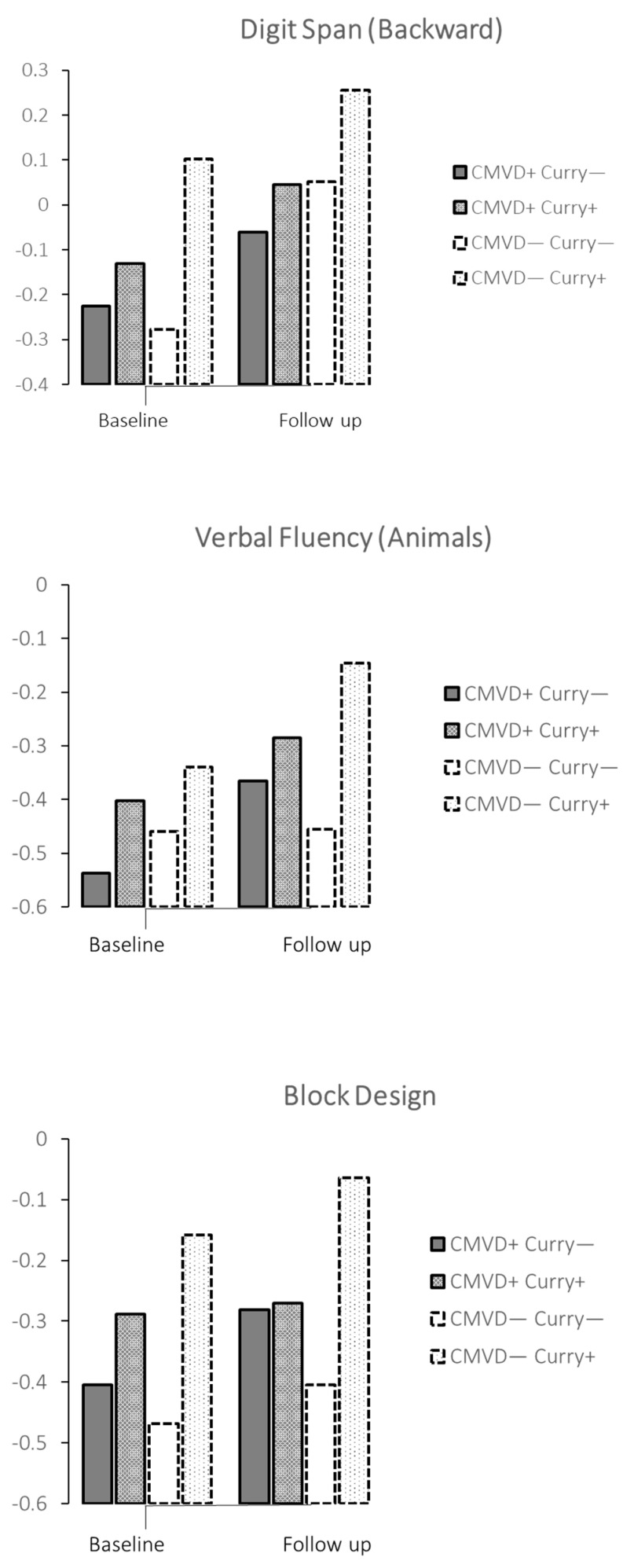
Significant associations between curry consumption and Digit Span (Backward), Verbal Fluency (Animals) and Block Design in subgroups of participants with and without cardiometabolic and vascular disease.

We further performed stratified analyses by subgroups of participants with or without cardiometabolic or cardiac diseases. (Table 4 and Figure 2) Among participants with cardiometabolic and cardiac diseases, curry consumption was associated with significantly higher performance on Digit Span (Backward) and Verbal Fluency (Animals). Among participants free of cardiometabolic and cardiac diseases, curry consumption was associated with significantly higher performance on Digit Span-Backward, Verbal Fluency-Animals and Block Design. The estimated Cohen’s d of effect size for curry consumption ranged from 0.098 (Digit Span-Backward) among those with cardiometabolic and cardiac disease to 0.305 (Block Design) among those without cardiometabolic and cardiac disease. 

## 4. Discussion

We found in this observational cohort study that the consumption of curcumin-rich curry in food was associated with the maintenance of higher levels of cognitive functioning over time among community-dwelling middle-aged and older Asian adults in Singapore. To our knowledge, this is the first longitudinal study demonstrating the cognitive benefits of curcumin from natural dietary food sources in humans. There are a number of notable observations from these findings for discussion.

*Cognitive domains*. Curcumin exposure from curry consumption appeared in this study to be associated with enhanced cognitive functioning on attention, short-term working memory, visual spatial constructional ability, language and executive function and evidently not with long-term memory ability. Non-amnestic related and executive function deficits are hallmarks of subcortical vascular cognitive impairment, a clinical continuum of vascular-related cognitive impairment, including vascular mild cognitive impairment and vascular dementia [38]. This suggests that, notwithstanding its known anti-amyloid, anti-tau, antioxidant and anti-inflammatory activities, curcumin exposure from dietary ingestion appears to offer neuroprotection especially through its beneficial metabolic, antiplatelet and cardioprotective properties. The results from the stratified analysis support this by showing that among participants with pre-existing metabolic and cardiovascular disease, curry consumption was associated with enhanced performance on attention, working memory and language executive function. In vitro and high-dose animal studies show that curcumin modulates molecular pathways that restore glucose homeostasis, lipid metabolism, endothelial function and insulin signaling and may thus offer potential cardiovascular protective effect. Clinical studies to date, however, remain rather unconvincing [2]. There was no suggestion from observing the cross-sectional relationship at baseline in this study that there was a meaningful association between curry consumption and cardiometabolic health, although the prevalence bias in this observation should be noted.

*Curcumin bioavailability*. The low bioavailability from oral ingestion of curcumin as a chemical compound with its poor aqueous solubility is well-known. This begs the question how the dietary ingestion of curcumin from natural food sources could actually make a difference in achieving desired physiological effects in various biological tissues, including the brain. Unlike pharmatherapeutic formulations, dietary curcumin is ingested not as curcumin per se, but as turmeric, which contains essential oils (turmeric oil) such as zingiberene, α-Turmerone and β-Turmerone [39]. These structural analogues of curcumin, together with spices and spice oils such as chilli, pepper, cumin, coriander and plant-based cooking oils, which are commonly blended with turmeric in the preparation of curry meals, as well as with intestinal bile salts, may act as its own lipid-based oral delivery systems, forming microemulsions, polymeric micelles or phospholipid complexes of curcumin, for enhanced solubility and bioavailability. There is evidence that the combination of piperine in black pepper with curcumin in turmeric increases the bioavailability of curcumin by 2000% and inhibits the intestinal and hepatic glucuronidation of curcumin [40].

*Microbiota–gut–brain axis.* High concentrations of curcumin remain in the intestines after oral ingestion. Bacterial enzymatic actions on curcumin may form metabolites pharmacologically more active than curcumin. A current working hypothesis is that curcumin could act primarily in the gastrointestinal tract, exerting direct regulatory effects on the brain in a bidirectional “gut–brain” communication axis, thereby influencing core neurological processes, including neurogenesis, synaptic plasticity, neurotransmitter signaling, neurodevelopment and neuroinflammation [41]. Studies show that by enhancing the diversity of the gut microbiota, curcumin and its metabolites restore gut microbial dysbiosis, decreases gut permeability and inflammation and metabolic endotoxemia associated with a high fat diet, obesity, metabolic syndrome, diabetes, CVD and neurodegenerative diseases [42,43]. These potential leads connecting dietary curcumin to the gut–brain axis development of cognitive disorders needs to be more intensively investigated in further studies.

*Dose–response relationship*. It is striking to note that better cognitive performance was evident with curry consumption frequencies that are relatively low in absolute terms. In this study, significantly better performance on the Digit Span (Backward) and Verbal Fluency (Animals) tasks were observed with curry consumption more frequent than only once a month (defined as “often” in this study). Performance on Block Design was even observed to be significantly better with curry consumption “occasionally” at less than once a month. Laboratory evidence suggests that curcumin is a hormetic agent (hormetin), exhibiting biphasic dose–responses, with low doses having stronger effects than high doses for some effects [44]. The hermetic effect of curcumin on cognitive functioning in humans is thus supported by the results in this study. The estimated Cohen’s d (between 0.10 and 0.30) indicated a small to medium effect. However, we estimated that the between-group differences in cognitive function were the equivalents of between 4 and 14 years of decrements in age.

*Strengths and limitations*. In this study, real-world observations of the potential cognitive benefits of curcumin in humans were provided in a large population-based cohort of community-dwelling older adults with follow-up over three to five years. This is relatively short compared to longer-term follow observation from, say, 10 years or more, and the changes in neurocognitive function were indeed very small. However, they reflect the actual observed amount of change associated with the exposure groups, as well as the survival bias from selective follow-up of participants with a relatively healthy risk profile and high cognitive performance status. The participants who were assessed at follow-up showed uniformly a more favorable baseline risk factor, curry consumption and neurocognitive profiles than those who were not assessed. The effect sizes are therefore probably underestimated, and this population selection bias thus warrants caution in generalizing the results from this study. The risk of neurocognitive disorders (mild cognitive impairment and dementia) was not analyzed as outcomes in this study and should be investigated in further studies.

*Health implications.* These observations suggest that population health in terms of healthy cognitive ageing and potential reduction of risks of dementia could be enhanced by increased consumption of curcumin in a turmeric-rich diet. The observed effect sizes were small, but nontrivial from a population health perspective. Further studies are desired to replicate these findings in other populations with measurable frequencies of turmeric consumption.

## Figures and Tables

**Table 1:** Baseline characteristics of SLAS participants (*N* = 2751) by curry consumption categories.

	Never/Rarely		<Once/Month		≥Once/Month to <Once/Week		≥Once/Week to Daily		*p*
*N* of participants		519		1306		559		367	
Sex: Male	31.2	(162)	33.8	(442)	39.4	(220)	48.2	(177)	<0.001
Age, years	67.9	±8.2	65.7	±7.1	65.2	±7.2	64.9	±7.3	<0.001
Ethnicity: Chinese	98.3	(510)	97.6	(1275)	95.5	(534)	84.5	(310)	<0.001
Non-Chinese	1.7	(9)	2.4	(31)	4.5	(25)	15.5	(57)	
Education: None	28.3	(147)	15.9	(208)	13.4	(75)	15.5	(57)	<0.001
1–6 years	42.4	(220)	39.4	(514)	38.3	(214)	33.2	(122)	
>6 years	29.3	(152)	44.7	(584)	48.3	(270)	51.2	(188)	
Smoking: Never	81.1	(421)	82.5	(1077)	81.9	(458)	77.4	(284)	0.216
Past smoker	11.8	(61)	9.7	(127)	10.7	(60)	11.2	(41)	
Current smoker	7.1	(37)	7.8	(102)	7.3	(41)	11.4	(42)	
Alcohol ≥ once/week	4.6	(24)	1.8	(24)	3.2	(18)	4.1	(15)	0.006
Physical activity score	2.31	1.73	2.42	1.61	2.37	1.78	2.44	1.85	0.618
Social activity score	3.07	2.65	3.19	2.58	3.43	2.66	3.20	2.59	0.146
Productive activity score	3.83	1.85	3.96	1.84	4.21	1.83	4.03	1.88	0.005
BMI, kg/m^2^	23.9	3.7	23.8	3.7	24.1	3.5	23.4	2.9	0.392
Central obesity	46.6	(242)	54.2	(708)	54.2	(303)	53.7	(197)	0.023
Hypertension	60.3	(313)	54.1	(706)	57.8	(323)	57.8	(212)	0.077
Diabetes or FBG > 5.6 mmol/L	25.0	(130)	26.3	(343)	28.1	(157)	26.2	(96)	0.723
High triglyceride	41.2	(214)	44.6	(583)	45.8	(256)	44.1	(162)	0.468
Low HDL-Cholesterol	44.9	(233)	49.4	(645)	49.7	(278)	47.1	(173)	0.297
Cardiac diseases	9.8	(51)	7.9	(103)	6.3	(35)	10.1	(37)	0.673
GDS depression score	1.45	2.25	0.88	1.64	1.03	2.08	1.29	2.40	<0.001

Continuous variables are normally distributed and expressed as mean ± SD. Categorical variables are expressed as % (N). *p*-values are based on ANOVA for continuous variables and chi-squared tests for categorical variables.

**Table 2:** Crude mean estimates of neurocognitive performance at baseline and follow-up by curry consumption levels.

Neurocognitive Performance	Curry Consumption	Baseline	Between Group ANOVA, *p*				Follow-Up			Between-Group ANOVA, *p*	
		*N*	Mean	±SE	Linear	Vs Never/Rarely	*n*	Mean	±SE	Linear	Vs Never/Rarely
MMSE	Never or rarely	516	27.1	±3.27	<0.001	Reference	272	27.7	3.18	<0.001	Reference
	Occasionally (<once a month)	1297	28.1	±2.40		<0.001	862	28.8	2.41		<0.001
	Often (>once a month, <once a week)	555	28.4	±1.86		<0.001	382	28.9	2.04		<0.001
	Very often (>once a week to daily	366	28.0	±2.70		<0.001	231	28.9	2.26		<0.001
Digit span (forward)	Never or rarely	478	0.07	±1.00	0.414	Reference	167	−0.03	1.02	0.316	Reference
	Occasionally (<once a month)	1108	0.05	±1.05		0.723	678	−0.01	1.05		0.847
	Often (>once a month, <once a week)	485	0.11	±1.07		0.551	293	−0.05	1.07		0.847
	Very often (>once a week to daily	324	−0.03	±1.06		0.159	169	−0.12	1.08		0.434
Digit span (backward)	Never or rarely	470	−0.24	±1.02	0.004	Reference	166	−0.11	0.93	0.007	Reference
	Occasionally (<once a month)	1098	−0.18	±1.00		0.289	674	0.05	0.98		0.062
	Often (>once a month, <once a week)	480	0.02	±1.05		<0.001	292	0.14	1.10		0.011
	Very often (>once a week to daily	317	−0.12	±1.06		0.117	169	0.17	1.05		0.011
RAVLT, delayed recall	Never or rarely	436	−0.34	±1.13	0.084	Reference	162	−0.14	1.09	0.985	Reference
	Occasionally (<once a month)	1060	−0.15	±1.02		0.002	676	−0.02	1.07		0.221
	Often (>once a month, <once a week)	465	−0.15	±1.01		0.006	288	−0.06	1.07		0.451
	Very often (>once a week to daily	299	−0.20	±1.10		0.084	169	−0.09	1.02		0.703
Visual Reproduction, delayed recall	Never or rarely	421	−0.25	±1.10	0.181	Reference	160	0.04	1.00	0.405	Reference
	Occasionally (<once a month)	1043	−0.15	±1.11		0.138	668	0.06	1.01		0.806
	Often (>once a month, <once a week)	465	−0.07	±1.05		0.019	289	0.13	1.09		0.367
	Very often (>once a week to daily	291	−0.18	±1.17		0.423	166	0.10	0.91		0.633
Verbal Fluency-Animals	Never or rarely	445	−0.27	±1.07	<0.001	Reference	167	−0.06	1.07	0.005	Reference
	Occasionally (<once a month)	1066	−0.16	±0.99		0.044	675	0.00	1.00		0.498
	Often (>once a month, <once a week)	458	0.00	±1.03		<0.001	292	0.11	1.03		0.086
	Very often (>once a week to daily	308	−0.05	±1.01		0.003	167	0.19	0.86		0.021
Trail-Making A	Never or rarely	281	−0.39	±1.44	0.005	Reference	159	0.10	1.38	0.013	Reference
	Occasionally (<once a month)	899	−0.09	±1.18		<0.001	663	0.01	1.16		0.389
	Often (>once a month, <once a week)	397	−0.12	±1.02		0.005	291	−0.06	1.32		0.180
	Very often (>once a week to daily	238	−0.17	±1.58		0.046	164	−0.21	0.82		0.019
Trail Making-B	Never or rarely	239	−0.32	±1.25	0.090	Reference	141	0.49	2.15	0.021	Reference
	Occasionally (<once a month)	825	−0.14	±1.20		0.056	630	0.15	1.46		0.014
	Often (>once a month, <once a week)	362	−0.11	±1.38		0.048	274	0.17	1.25		0.035
	Very often (>once a week to daily	218	−0.11	±1.24		0.077	157	0.00	1.13		0.004
Block Design	Never or rarely	324	−0.38	±1.13	0.002	Reference	162	−0.18	1.00	0.082	Reference
	Occasionally (<once a month)	886	−0.18	±1.03		0.004	671	−0.05	0.99		0.120
	Often (>once a month, <once a week)	343	−0.06	±1.03		<0.001	288	0.02	0.99		0.035
	Very often (>once a week to daily	232	−0.14	±1.11		0.010	166	−0.01	0.92		0.113

**Table 3:** Mixed linear model analyses: covariate-adjusted mean neurocognitive z-scores at baseline and follow-up by curry consumption.

Neurocognitive Performance	Curry Consumption		Baseline Mean ± SE		Follow-Up Mean ± SE		Group	*p*	δ	95%	CI	Cohen’s d
MMSE	Never or rarely	A	26.9	±0.198	25.6	±0.207	Overall	0.284				
	Occasionally (<once a month)	B	27.1	±0.193	25.7	±0.200	B vs. A	0.141	0.124	−0.041,	0.289	0.048
	Often (>once a month, <once a week)	C	27.2	±0.204	25.7	±0.213	C vs. A	0.062	0.186	−0.010,	0.382	0.072
	Very often (>once a week to daily	D	27.0	±0.203	25.8	±0.217	D vs. A	0.314	0.115	−0.109,	0.339	0.045
Digit span (forward)	Never or rarely	A	−0.23	±0.108	−0.38	±0.119	Overall	0.713				
	Occasionally (<once a month)	B	−0.30	±0.106	−0.40	±0.111	B vs. A	0.299	0.048	−0.139,	0.043	0.046
	Often (>once a month, <once a week)	C	−0.25	±0.111	−0.37	±0.120	C vs. A	0.906	0.006	−0.114,	0.101	0.006
	Very often (>once a week to daily)	D	−0.28	±0.110	−0.38	±0.124	D vs. A	0.703	0.024	−0.147,	0.099	0.023
Digit span (backward)	Never or rarely	A	−0.23	±0.105	−0.06	±0.114	Overall	0.012				
	Occasionally (<once a month)	B	−0.23	±0.103	−0.03	±0.108	B vs. A	0.700	0.017	−0.070,	0.105	0.017
	Often (>once a month, <once a week)	C	−0.17	±0.108	−0.05	±0.115	C vs. A	0.010	0.135	0.032,	0.239	0.132
	Very often (>once a week to daily)	D	−0.12	±0.106	0.09	±0.119	D vs. A	0.028	0.133	0.014,	0.252	0.130
RAVLT, delayed recall	Never or rarely	A	−0.44	±0.113	−0.30	±0.123	Overall	0.156				
	Occasionally (<once a month)	B	−0.32	±0.111	−0.23	±0.115	B vs. A	0.041	0.096	0.004,	0.189	0.091
	Often (>once a month, <once a week)	C	−0.34	±0.115	−0.22	±0.124	C vs. A	0.118	0.087	−0.022,	0.196	0.083
	Very often (>once a week to daily)	D	−0.27	±0.114	−0.23	±0.127	D vs. A	0.069	0.117	−0.009,	0.242	0.111
VR, delayed recall	Never or rarely	A	−0.42	±0.116	−0.16	±0.123	Overall	0.303				
	Occasionally (<once a month)	B	−0.42	±0.113	−0.25	±0.117	B vs. A	0.335	−0.045	−0.137,	0.047	0.041
	Often (>once a month, <once a week)	C	−0.35	±0.118	−0.14	±0.124	C vs. A	0.422	0.044	−0.064,	0.153	−0.040
	Very often (>once a week to daily)	D	−0.37	±0.118	−0.20	±0.126	D vs. A	0.859	0.011	−0.114,	0.137	−0.010
Verbal Fluency-Animals	Never or rarely	A	−0.57	±0.108	−0.43	±0.117	Overall	0.017				
	Occasionally (<once a month)	B	−0.59	±0.106	−0.45	±0.110	B vs. A	0.705	−0.017	0.103,	0.070	0.016
	Often (>once a month, <once a week)	C	−0.47	±0.111	−0.37	±0.118	C vs. A	0.112	−0.083	0.019,	0.185	0.081
	Very often (>once a week to daily)	D	−0.46	±0.109	−0.28	±0.121	D vs. A	0.026	0.160	0.134,	0.251	0.131
Trail-Making A	Never or rarely	A	−0.61	±0.178	−0.53	±0.181	Overall	0.375				
	Occasionally (<once a month)	B	−0.41	±0.172	−0.31	±0.174	B vs. A	0.315	0.060	−0.057,	0.176	0.047
	Often (>once a month, <once a week)	C	−0.42	±0.176	−0.43	±0.180	C vs. A	0914	−0.007	−0.143,	0.128	0.006
	Very often (>once a week to daily)	D	−0.46	±0.176	−0.49	±0.182	D vs. A	0.496	−0.055	−0.214,	0.104	0.044
Trail Making-B	Never or rarely	A	−0.64	±0.198	−0.20	±0.209	Overall	0.975				
	Occasionally (<once a month)	B	−0.52	±0.191	−0.19	±0.198	B vs. A	0.071	0.008	−0.131,	0.148	0.007
	Often (>once a month, <once a week)	C	−0.49	±0.195	−0.29	±0.208	C vs. A	0.083	−0.023	−0.186,	0.139	0.019
	Very often (>once a week to daily)	D	−0.45	±0.194	−0.31	±0.212	D vs. A	0.097	−0.013	−0.203,	0.176	0.011
Block Design	Never or rarely	A	−0.48	±0.142	−0.42	±0.146	Overall	0.003				
	Occasionally (<once a month)	B	−0.42	±0.140	−0.29	±0.141	B vs. A	0.039	0.096	0.005,	0.188	0.090
	Often (>once a month, <once a week)	C	−0.28	±0.146	−0.22	±0.146	C vs. A	<0.001	0.198	0.089,	0.308	0.186
	Very often (>once a week to daily)	D	−0.30	±0.145	−0.27	±0.147	D vs. A	0.010	0.166	0.040,	0.292	0.156

Covariables in model: age, sex, ethnicity (Chinese versus non-Chinese), educational level, leisure time activity score, smoking (nonsmoker, ex-smoker, current smoker), alcohol (never, <once/week, ≥once/week), central obesity, hypertension, high fasting blood glucose or diabetes, high triglyceride, low HDL-cholesterol, cardiac diseases and GDS. MMSE: Time, *p* ≤ 0.001, Group × time, *p* = 0.458; Digit span (forward): time, *p* = 0.001, group × time, *p* = 0.950; Digit span (backward): time, *p* ≤ 0.001, group × time, *p* = 0.849 RAVLT, delayed recall: time, *p* = 0.012, group × time, *p* = 0.835; Visual Reproduction, delayed recall: time, *p* = 0.001, group × time, *p* = 0.759; Verbal Fluency-Animals: time, *p* = 0.001, group × time, *p* = 0.937; Trail-Making A: time, *p* = 0.027, group × time, *p* = 0.130; Trail Making-B: time, *p* = 0.001, group × time, *p* = 0.107; Block Design: time, *p* = 0.087, group × time, *p* = 0.803.

**Table 4:** Mixed linear model analyses: covariate-adjusted mean neurocognitive z-scores at baseline and follow-up by cardiometabolic morbidity and curry consumption groups.

Neurocognitive Test	Curry > Once/Month to Daily		Baseline		Follow-Up		Group	*p*	δ	95%	CI	Cohen’s d
			Mean	95% CI	Mean	95% CI						
Cardiometabolic or cardiac diseases												
MMSE	No	A	26.9	26.6, 27.3	27.5	27.2, 27.9						
	Yes	B	27.1	27.0, 27.4	27.6	27.2, 28.0	A vs. B	0.213	0.095	−0.054,	0.244	0.037
Digit span (forward)	No	A	−0.24	−0.44, −0.05	−0.37	−0.58, −0.17						
	Yes	B	−0.25	−0.45, −0.05	−0.43	−0.64, −0.21	A vs. B	0.450	−0.031	−0.110,	0.049	0.029
Digit span (backward)	No	A	−0.23	−0.41, −0.04	−0.06	−0.26, 0.14						
	Yes	B	−0.13	−0.32, 0.06	−0.05	−0.16, 0.25	A vs. B	0.010	0.101	0.024,	0.178	0.098
RAVLT, delayed recall	No	A	−0.35	−0.56, −0.14	−0.22	−0.44, −0.01						
	Yes	B	−0.29	−0.49, −0.98	−0.22	−0.44, 0.00	A vs. B	0.387	0.036	−0.045,	0.117	0.034
VR, delayed recall	No	A	−0.47	−0.68, −0.26	−0.23	−0.45, −0.02						
	Yes	B	−0.38	−0.59, −0.17	−0.21	−0.43, 0.01	A vs. B	0.202	0.052	−0.028,	0.132	0.047
Verbal Fluency-Animals	No	A	−0.54	0.73, −0.34	−0.37	−0.57, −0.16						
	Yes	B	−0.40	−0.60, −0.20	−0.28	−0.49, −0.07	A vs. B	0.005	0.108	0.032,	0.184	0.106
Trail-Making A	No	A	−0.54	−0.87, −0.22	−0.32	−0.65, 0.00						
	Yes	B	−0.49	−0.81, −0.17	−0.44	−0.76, −0.11	A vs. B	0.558	−0.030	−0.129,	0.070	0.024
Trail Making-B	No	A	−0.49	−0.85, −0.12	−0.05	−0.42, 0.32						
	Yes	B	−0.41	−0.77, −0.05	−0.07	−0.45, 0.30	A vs. B	0.661	0.027	−0.093,	0.146	0.021
Block Design	No	A	−0.40	−0.66, −0.14	−0.28	−0.54, −0.02						
	Yes	B	−0.29	−0.55, −0.02	−0.27	−0.53, −0.01	A vs. B	0.119	0.063	−0.016,	0.143	0.060
No cardiometabolic or cardiac diseases												
MMSE	No	C	27.0	26.2, 27.1	27.3	26.8, 27.8						
	Yes	D	26.5	26.0, 27.0	27.2	26.6, 27.7	C vs. D	0.396	−0.158	−0.522,	0.207	0.061
Digit span (forward)	No	C	−0.30	−0.53, −0.06	−0.19	−0.46, 0.07						
	Yes	D	−0.20	−0.46, 0.07	−0.28	−0.59, 0.04	C vs. D	0.919	0.010	−0.185,	0.205	0.010
Digit span (backward)	No	C	−0.28	−0.50, −0.05	0.05	−0.20, 0.30						
	Yes	D	−0.10	−0.16, 0.36	0.26	−0.05, 0.56	C vs. D	0.003	0.291	0.101,	0.480	0.284
RAVLT, delayed recall	No	C	−0.22	−0.47, 0.03	−0.11	−0.38, 0.16						
	Yes	D	−0.33	−0.461, 0.05	−0.14	−0.47, 0.18	C vs. D	0.508	−0.068	−0.269,	0.133	0.064
VR, delayed recall	No	C	−0.39	−0.64, −0.13	−0.34	−0.61, −0.07						
	Yes	D	−0.48	−0.77, −0.19	−0.06	−0.38, 0.25	C vs. D	0.346	0.095	−0.103,	0.294	0.086
Verbal Fluency-Animals	No	C	−0.46	−0.69, −0.22	−0.46	−0.71, −0.20						
	Yes	D	−0.34	−0.61, −0.07	−0.15	−0.45, −0.16	C vs. D	0.024	0.215	0.028,	0.402	0.211
Trail-Making A	No	C	−0.49	−0.87, −0.11	−0.35	−0.73, 0.02						
	Yes	D	−0.41	−0.84, 0.02	−0.67	−1.10, 0.24	C vs. D	0.372	−0.118	−0.376,	0.141	0.093
Trail Making-B	No	C	−0.33	−0.74, 0.08	−0.14	−0.58, 0.31						
	Yes	D	−0.30	−0.76, 0.16	−0.52	−1.03, 0.00	C vs. D	0.255	−0.176	−0.478,	0.127	0.139
Block Design	No	C	−0.47	−0.78, −0.16	−0.40	−0.70, −0.10						
	Yes	D	−0.16	−0.53, 0.22	−0.06	−0.40, 0.27	C vs. D	0.003	0.325	0.110,	0.541	0.305

Covariables in the model: age, sex, ethnicity (Chinese versus non-Chinese), educational level, leisure time activity score, smoking (nonsmoker, ex-smoker, current smoker), alcohol (never, <once/week, ≥once/week), central obesity, hypertension, high fasting blood glucose or diabetes, high triglyceride, low HDL-cholesterol, cardiac diseases and GDS score. Hypothesis-driven pairwise comparisons of interest are shown for A vs. B and C vs. D. MMSE: Group *p* = 0.005, Tim, *p* ≤ 0.001, Group × time, *p* = 0.949; Digit span (forward): group, *p* = 0.432, time, *p* = 0.191, group × time, *p* = 0.193; Digit span (backward): group, *p* ≤ 0.001, time, *p* ≤ 0.001, group × time, *p* = 0.581; RAVLT, delayed recall: group, *p* = 0.278, time, *p* = 0.028, group × time, *p* = 0.869; Visual Reproduction, delayed recall: group, *p* = 0.474, time, *p* = 0.001, group × time, *p* = 0.235; Verbal Fluency-Animals: group, *p* = 0.004, time, *p* = 0.018, group × time, *p* = 0.517; Trail-Making A: group, *p* = 0.756, time, *p* = 0.604, group × time, *p* = 0.090; Trail Making-B: group, *p* = 0.649, time, *p* = 0.023, group × time, *p* = 0.064 Block Design: group, *p* = 0.011, time, *p* = 0.198, group × time, *p* = 0.610.

## Data Availability

The datasets used and/or analyzed during the current study are available from the corresponding author on reasonable request.

## Supplementary Materials

The following supporting information can be downloaded at: https://www.mdpi.com/article/10.3390/nu14061189/s1, Table S1: Association of known or potential risk factors with neurocognitive test performance.
