# Supplementary material for: Curcumin-Rich Curry Consumption and Neurocognitive Function from 4.5-Year Follow-Up of Community-Dwelling Older Adults (Singapore Longitudinal Ageing Study)
Source: Nutrients. 2022 Mar 11;14(6):1189. doi: 10.3390/nu14061189 (PMC8952785; doi:10.3390/nu14061189)
Supplement: Supplementary file 1 [file nutrients-14-01189-s001.zip › nutrients-1572018-supplementary.pdf]

Supplementary Table S1. Association of known or potential risk factors with neurocognitive test performance.

	**MMSE**		**DS-F**		**DS-B**		RAVLT(DR)		VR (DR)		VF (A)		TMT-A		TMT-B		BD	
	**Beta**	***p***	**Beta**	***p***	**Beta**	***p***	Beta	*p*	Beta	*p*	Beta	*p*	Beta	*p*	Beta	*p*	Beta	*p*
Age	−0.195	0.000	0.042	0.060	−0.021	0.340	−0.042	0.063	−0.053	0.022	−0.024	0.280	−0.149	0.000	−0.096	0.000	−0.144	0.000
Sex	−0.074	0.000	−0.071	0.005	−0.109	0.000	0.108	0.000	−0.024	0.351	−0.164	0.000	−0.179	0.000	−0.191	0.000	−0.182	0.000
Ethnicity	−0.062	0.000	−0.139	0.000	−0.091	0.000	−0.042	0.039	−0.071	0.001	−0.064	0.001	−0.100	0.000	−0.093	0.000	−0.107	0.000
Education	0.358	0.000	0.062	0.006	0.096	0.000	0.079	0.001	0.090	0.000	0.170	0.000	0.024	0.337	0.048	0.066	0.041	0.110
Fitness activity	0.048	0.005	−0.016	0.436	−0.007	0.727	−0.020	0.355	0.031	0.154	0.042	0.046	0.033	0.174	0.012	0.627	0.043	0.075
Social activity	0.003	0.884	0.061	0.004	0.056	0.009	0.056	0.010	0.071	0.001	0.012	0.564	0.090	0.000	0.091	0.000	0.092	0.000
Productive activity	0.122	0.000	−0.005	0.820	0.011	0.632	0.103	0.000	0.073	0.002	0.103	0.000	0.079	0.002	0.022	0.402	0.056	0.026
Smoking	0.033	0.067	−0.004	0.863	0.071	0.002	−0.023	0.310	−0.033	0.155	−0.042	0.059	−0.061	0.018	−0.039	0.146	−0.042	0.106
Alcohol	−0.044	0.007	−0.002	0.931	0.040	0.050	0.020	0.320	−0.003	0.879	−0.010	0.620	−0.041	0.071	−0.035	0.145	0.014	0.528
BMI	−0.013	0.519	−0.089	0.001	−0.010	0.691	0.000	0.986	0.026	0.333	0.046	0.077	−0.049	0.094	−0.040	0.193	0.025	0.385
Central obesity	0.053	0.011	0.044	0.089	−0.015	0.576	−0.005	0.854	−0.058	0.031	−0.030	0.251	0.055	0.060	0.010	0.741	−0.018	0.536
Hypertension	−0.005	0.761	−0.030	0.136	0.006	0.775	0.009	0.676	−0.008	0.715	−0.005	0.796	−0.001	0.952	−0.007	0.762	−0.002	0.922
Diabetes	−0.002	0.895	−0.015	0.482	0.018	0.392	−0.047	0.025	−0.012	0.563	−0.007	0.718	0.027	0.246	−0.061	0.013	−0.018	0.444
High TG	0.033	0.118	−0.026	0.331	−0.014	0.609	0.014	0.592	0.025	0.360	0.075	0.004	0.044	0.141	−0.003	0.916	0.016	0.597
Low HDL	−0.010	0.630	0.038	0.155	0.018	0.512	−0.005	0.839	0.009	0.748	−0.052	0.050	−0.012	0.697	0.020	0.532	0.039	0.194
CVD	−0.031	0.064	−0.008	0.711	−0.004	0.847	−0.046	0.028	−0.022	0.310	−0.042	0.042	0.014	0.557	−0.043	0.082	−0.033	0.154
GDS	−0.128	0.000	−0.035	0.079	−0.018	0.374	−0.069	0.001	−0.058	0.005	−0.010	0.612	−0.016	0.468	−0.003	0.906	−0.074	0.001
